# The influence of dietary diversity on the nutritional status of children between 6 and 23 months of age in Tanzania

**DOI:** 10.1186/s12887-019-1897-5

**Published:** 2019-12-28

**Authors:** Ahmed Gharib Khamis, Akwilina Wendelin Mwanri, Julius Edward Ntwenya, Katharina Kreppel

**Affiliations:** 1 Department of Epidemiology and Biostatistics, Muhimbili University of Health and Allied Sciences, Dar-es-Salaam, Tanzania; 2 Department of Food Technology, Nutrition and Consumer Sciences, Sokoine University of Agriculture, P. O Box 3006, Chuo Kikuu, Morogoro, Tanzania; 3 Department of Public Health, The University of Dodoma, P.O. Box 395, Dodoma, Tanzania; 4 School of Life Sciences and Bio-Engineering, Nelson Mandela African Institution of Science and Technology, Arusha, Tanzania; 5 Department of Environmental Health and Ecological Sciences, Ifakara Health Institute, Dar-es-Salaam, Tanzania

**Keywords:** Dietary diversity, Complementary feeding, Undernutrition, Pediatric, Infants and young children, Tanzania

## Abstract

**Background:**

Undernutrition poses a serious health challenge in developing countries and Tanzania has the highest undernutrition burden of Eastern and Southern Africa. Poor infant and young child feeding practices have been identified as the main causes for undernutrition. As dietary diversity is a major requirement if children are to get all essential nutrients, it can thus be used as one of the core indicators when assessing feeding practices and nutrition of children. Therefore, adequate information on the association between dietary diversity and undernutrition to identify potential strategies for the prevention of undernutrition is critical. Here we examined to what extent dietary diversity is associated with undernutrition among children of 6 to 23 months in Tanzania.

**Methods:**

Using existing data from the Tanzania Demographic and Health Survey of 2015–2016, we carried out secondary data analysis. Stunting, Wasting and Underweight of the surveyed children were calculated from Z-scores of Height-for-age (HAZ), Weight-for-height (WHZ) and Weight-for-age (WAZ) based on 2006 WHO standards. A composite dietary diversity score was created by summing the number of food groups eaten the previous day as reported for each child by the mother ranging from 0 to 7. Then, minimum dietary diversity (MDD) of 4 food groups out of seven was used to assess the diversity of the diet given to children. Bivariate and multivariate logistic regression techniques were used to assess the crude and adjusted odds ratios of stunting, wasting and being underweight.

**Results:**

A total of 2960 children were enrolled in this study. The prevalence of stunting was 31%, wasting 6% and underweight 14%. Among all children, 51% were female and 49% male. The majority (74%) of children did not reach the MDD. The most commonly consumed types of foods were grains, roots and tubers (91%), and Vitamin A containing fruits and vegetables (65%). The remaining food groups were reported to be consumed by a much lower proportion of children, including eggs (7%), meat and fish (36%), milk and dairy products (22%), as well as legumes and nuts (35%), and other vegetables (21%). Consumption of a diverse diet was significantly associated with a reduction of stunting, wasting and being underweight in children. The likelihood of being stunted, wasted and underweight was found to decrease as the number of food groups consumed increased. Children who did not receive the MDD had a significantly higher likelihood of being stunted (AOR = 1.37, 95% CI; 1.13–1.65) and underweight (AOR = 1.49, 95% CI; 1.15–1.92), but this was not the case for wasting. Consumption of animal-source foods has been found to be associated with reduced stunting among children.

**Conclusion:**

Consumption of a diverse diet is associated with a reduction in undernutrition among children of 6 to 23 months in Tanzania. Measures to improve the type of complementary foods in order to meet the energy and nutritional demands of children should be considered in Tanzania.

## Background

Undernutrition poses a serious challenge in developing countries [1]. The World Health Report of 2016 estimated that about 155 million children under five years of age are stunted and 52 million wasted [2]. The rates of undernutrition were substantially higher in the Sub-Saharan African (SSA) region [1, 3]. In Tanzania, a high prevalence of chronic and acute undernutrition still persists. It was estimated that about 450,000 children in Tanzania are acutely undernourished or wasted, with over 100,000 suffering from the most severe form of acute malnutrition [4], making it a country with one of the highest undernutrition burdens in Eastern and Southern Africa [5]. The recently released national survey of 2018 reported that 31.8% of children were stunted, 3.6% were wasted and 14% were underweight [6]. The damage caused by undernutrition during the first two years of life may compromise cognitive development of the children and in turn result in poor educational achievement, low economic productivity, and is associated with illness and mortality during adulthood [7].

Poor infant and young child feeding practices are the main causes of undernutrition in Tanzania [8, 9] and other developing countries [2]. From the age of 6 months, breastfeeding is no longer able to meet all nutritional requirements of a growing child, and therefore, the consumption of adequate, diversified food is necessary [10, 11]. Globally, however, only less than one-fourth of infants aged 6–23 months meet the recommended criteria for dietary diversity, and only a few of them are receiving a nutritionally adequate diet [2]. The World Health Organization has recommended that an infant should receive the minimum dietary diversity (MDD) of at least four food groups out of seven in order to maintain proper growth and development during this critical period [12], but many children cannot meet these criteria. In Tanzania for example, according to the recent report, only 35.1% of children aged 6 to 23 months had received the MDD [13], and only 23.3% in Ethiopia [14]. Dietary diversity, as a marker of micronutrient adequacy, may increase nutrient density of the complementary foods [15], which promote optimal child growth and development. Receiving an inadequately diversified diet may lead to undernutrition [16], and predispose children to opportunistic infections and severe illnesses [7].

Although the association between dietary diversity and the nutritional status of children has already been studied in various countries [16–21], studies that use large-scale data are scarce, particularly in Tanzania. In addition, many previous studies in Tanzania have largely focused on other aspects of child nutrition, like micronutrient content [9], complementary feeding practices [9, 22–25] and their determinants [26, 27]. None of them, according to our literature review, has examined the association between child diet diversity and nutritional status. Understanding the influence of dietary diversity on the nutritional status of children could be useful to inform nutrition policy and propose interventions that focus on improving the quality of complementary foods. Findings from this study will therefore be important to public health experts in Tanzania and help to work towards the Sustainable Development Goal-2 (SDG-2) agenda, which aims to end malnutrition in all its forms by 2030 [28]. The present study examined the role of child dietary diversity on undernutrition in Tanzania by using the large available dataset, which is representative of the whole country.

## Methods

### Data source

This is a secondary data analysis from data of the Tanzania Demographic and Health Survey (TDHS) 2015–2016 which was implemented by the National Bureau of Statistics in collaboration with other government partners [13]. Data collection procedures have been described and published in the TDHS 2015 report [13]. Briefly, TDHS of 2015 was designed to produce representative samples at national, regional and rural-urban levels. The TDHS of 2015 was part of the worldwide demographic and health survey program, aimed at assisting countries to collect data to monitor and evaluate their population, health and nutrition programs. The survey employed a two-stage sampling design. In the first-stage, 608 clusters were randomly selected. The second-stage sampling involved systematic sampling of 22 households from the selected clusters. A total of 13,376 households were included. In this analysis, only data of children aged between 6 to 23 months during the study, matched with their mothers, were finally selected for further analysis.

**Fig. 1:**
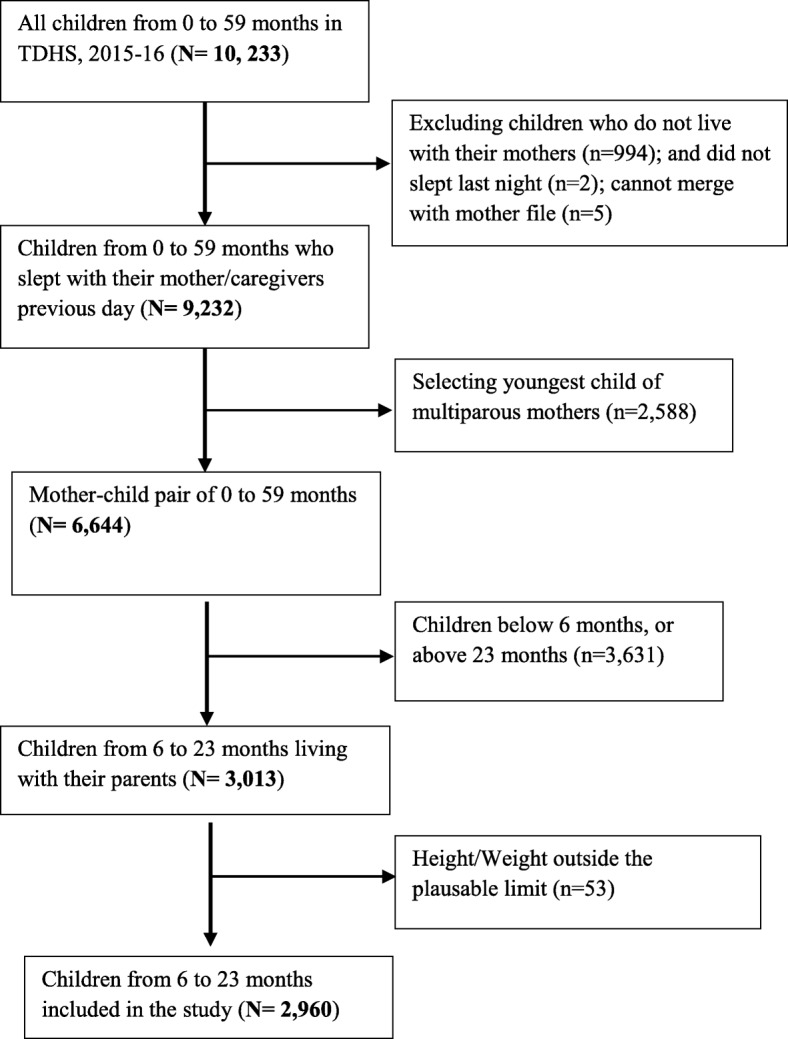
Selection of children of 6 to 23 months from TDHS 2015-16

We used the Kids file (KR file) and Mother file (MR file) from the TDHS data obtained from an online data repository to get information about nutrition status and dietary diversity of children [20]. From 10,233 children of 0–59 month old assessed in the survey, we selected 2960 younger children aged between 6 and 23 months living with their mothers. This was because in the TDHS, information on feeding practices was collected only for the youngest child of this age group. Details of the procedure used to select children for this study are described in Fig. 1.

### Nutrition status calculation

Weight and height were recorded by directly measuring children to generate anthropometric variables used in this study [29]. Stunting, wasting and underweight were calculated from height-for-age z-scores (HAZ), weight-for-height z-scores (WHZ), and weight-for-age z-scores (WAZ); based on 2006 WHO standards [29]. Height-for-age z-scores are used to measure chronic malnutrition due to prolonged food deprivation. Weight-for-height z-scores captures undernutrition due to recent food deprivation and malnutrition, and WAZ measures the child’s body mass relative to its chronologic age, and used as a proxy for underweight. Children whose HAZ, WHZ and WAZ was below minus two standard deviations (− 2 SD) from the median of the reference population were considered short for their age (stunted), wasted and underweight respectively [29]. We excluded missing values and biologically implausible values in our study as shown in Fig. 1.

### Dietary diversity calculation

To measure dietary diversity, we adopted the WHO’s Infant and Young Children Feeding guidelines (IYCF) [12] as an internationally acceptable complementary feeding guideline. The dataset used in the analysis contains information on food items used to calculate the diet diversity indicator. The TDHS survey collected information on food items a child consumed throughout the previous day. We categorized these food items into seven major food groups based on the WHO’s IYCF guidelines. These food groups are: (i) grains, roots, and tubers; (ii) legumes and nuts; (iii) flesh foods (meat, fish, poultry and liver/organ meats); (iv) eggs; (v) vitamin A rich fruits and vegetables; (vi) dairy products (milk, yogurt, cheese); (vii) other fruits and vegetables. If a child consumed at least one food item from a food group throughout the previous day, the group was assigned a value of one (1) for that child, and zero (0) if not consumed. The group scores are then summed up to obtain the dietary diversity score, which ranges from zero to seven, whereby zero represents non-consumption of any of the food items in the food groups, and seven represents the highest level of diet diversification. The MDD was attained if a child had consumed four or more food groups (FG ≥ 4) out of the seven food groups over the previous day.

### Other covariates

Based on the reviewed literature and the objective of this study we considered other characteristics of the child reported by the mother. A child’s characteristics included in the study were age, gender, and its size at birth as perceived subjectively by the mother, as well as place of birth and any presence of fever and diarrhea in the past two weeks before the interview [18, 21].

### Statistical analysis

We presented categorical variables as proportions, and continuous variables as means and standard deviation (Means ± SD). To determine the relationship between dietary diversity and nutrition status outcomes, i.e. stunting, wasting and underweight, we first built a series of bivariate logistic regression (for dichotomous outcomes) models. Dietary diversity score, the independent variable of interest, was captured as a continuous variable from the count of the number of food groups the child consumed the previous day before the survey. A separate model was created for each anthropometric outcome, with dietary diversity scores as the independent variable. In addition, the same was done for the MDD as a categorical indicator. Each food group was entered in a bivariate and multivariate model to test its association with nutrition status of the children. The multivariable models for each outcome were controlled for the child’s gender, age, presence of fever (yes/no), and diarrhea (yes/no) in the past two weeks before the survey [18, 21]. Results were considered significant if *p* < 0.05. All analyses were performed using Statistical Package for the Social Science (SPSS) software version 23, Stata software, and graphically visualized in Excel.

## Results

### Characteristics of the children

Table 1 presents the general characteristics of the 2960 children enrolled in this study. Among them, 51 and 49% were female and male respectively. Their mean (SD) age was 14.2 (5.1) months with about 64 and 34% born at health facility and at home, respectively. Among the studied children, 31% were stunted, 6% wasted and 14% underweight. More than 60% had normal birth weight of above 2.5 kg. About 79% of children had no diarrhea within two weeks before the survey, and 78% had no sign of fever.

**Table 1:** Descriptive characteristics of the children aged 6–23 months included in the study (*N* = 2960)

Variable	Mean (SD) and Frequency (%*)*
Age of Children (months)	*14.2 (SD = 5.1 months)*
Prevalence of undernutrition	
Stunting (HAZ < -2SD)	927 (31%)
Wasting (WHZ < -2SD)	179 (6%)
Underweight (WAZ < -2SD)	423 (14%)
Gender	
Male	1459 (49%)
Female	1501 (51%)
Place of Birth	
Health facility	1913 (64%)
At home	1001 (34%)
Other places	46 (2%)
Size at Birth	
Above 2.5 kg	1801 (61%)
Below 2.5 kg	100 (3%)
Not reported	1059 (36%)
Presence of Diarrhea	
YES	625 (21%)
NO	2335 (79%)
Presence of Fever	
YES	651 (22%)
NO	2309 (78%)

*HAZ Height-for-Age Z-scores, *WHZ* Weight-for-Height Z-scores, *WAZ* Weight-for-Age Z-scores, *SD* Standard deviation.

### Dietary diversity of 6 to 23 month old children

**Fig. 2:**
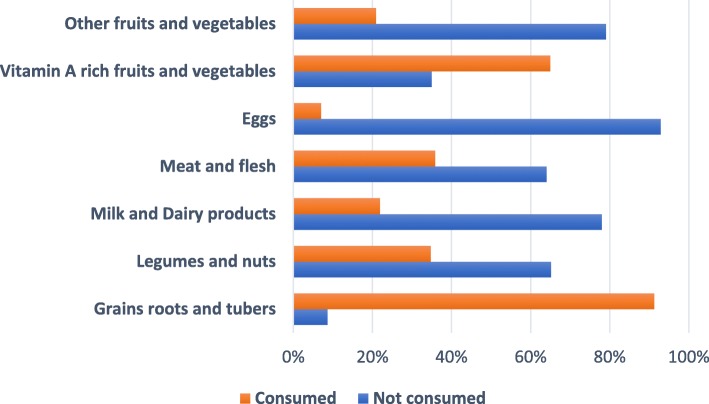
Proportion of children in terms of their food consumption

**Fig. 3:**
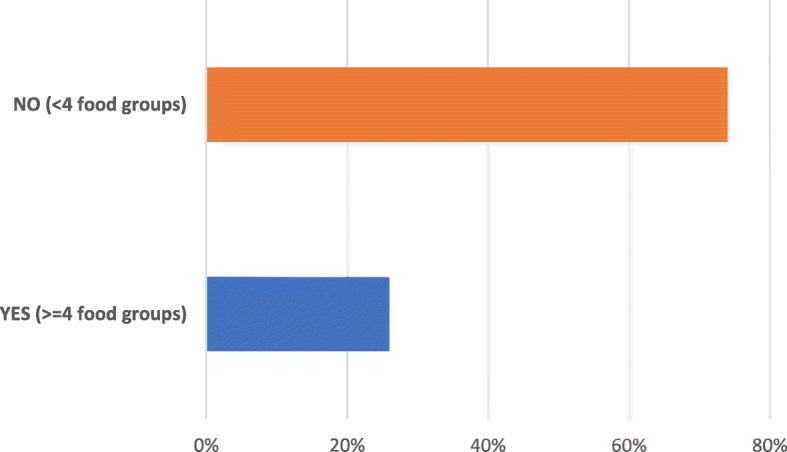
Proportion of children reaching the recommended minimum dietary diversity

Fig. 2 shows the proportion of children in terms of food groups consumed in the previous day before the survey. This analysis found that the majority (74%) of children did not meet the recommended MDD of more than 4 food groups the previous day. Only (26%) of them had received a diversified diet (Fig. 3). The most commonly consumed types of foods were grains, roots and tubers (91%) and vitamin A containing fruits and vegetables (65%). Lesser proportions of children were reported to have consumed food items of the remaining food groups including eggs (7%), meat and fish (36%), milk and dairy products (22%), as well as legumes and nuts (35%) and other vegetables (21%).

### Association between dietary diversity and undernutrition

Table 2 presents the results of an association between dietary diversity and stunting, wasting and underweight. Based on this analysis using the dietary diversity score and MDD as independent variables, consumption of a diverse diet was significantly associated with a reduction in stunting, wasting and being underweight for children. The likelihood of suffering from stunting, wasting and underweight was found to decrease as the number of food groups consumed increased. Therefore, both the dietary diversity score and MDD analysis showed that children who consumed diverse diets are less likely to be undernourished than those who have a less diverse diet. In an adjusted model, children who did not receive the MDD had a significantly higher likelihood of becoming stunted (AOR = 1.37, 95% CI; 1.13–1.65) and underweight (AOR = 1.49, 95% CI; 1.15–1.92) compared to children who received the MDD. However; there was no association between the consumption of the MDD and wasting (unadjusted: OR = 1.19, 95% CI 0.83–1.71; adjusted: AOR = 1.18, 95% CI; 0.82–1.69).

**Table 2:** Bivariate and multivariate logistic regression analysis of the association between dietary diversity and stunting, wasting and underweight

	Stunting (HAZ < −2SD)			Wasting (WHZ < −2SD)			Underweight (WAZ < −2SD)		
Diversity score	*n*	OR (95% CI)	AOR^a^ (95% CI)	*n*	OR (95% CI)	AOR^a^ (95% CI)	*n*	OR (95% CI)	AOR^a^ (95% CI)
0	18	Ref.	Ref.	8	Ref.	Ref.	11	Ref.	Ref.
1	93	0.8 (0.43–1.47)	0.65 (0.35–1.23)	29	0.56 (0.24–1.3)	0.5 (0.21–1.18)	54	0.7 (0.37–1.6)	0.67 (0.32–1.39)
2	285	0.94 (0.53–1.69)	0.57 (0.31–1.05)	50	0.34 (0.15–0.76)**	0.3 (0.13–0.7)**	134	0.69 (0.35–1.38)	0.52 (0.26–1.06)
3	311	1.08 (0.61–1.94)	0.61 (0.33–1.12)	51	0.35 (0.15–0.78)*	0.32 (0.14–0.73)**	136	0.71 (0.35–1.41)	0.52 (0.26–1.05)
4	166	0.91 (0.5–1.64)	0.49 (0.26–0.91)*	31	0.35 (0.15–0.82)*	0.32 (0.13–0.76)*	67	0.56 (0.27–1.14)	0.4 (0.19–0.83)*
5	46	0.68 (0.35–1.31)	0.36 (0.18–0.72)**	9	0.3 (0.11–0.82)*	0.26 (0.09–0.74)*	19	0.46 (0.20–1.04)	0.32 (0.14–0.74)**
6	6	0.44 (1.55–1.25)	0.25 (0.85–0.72)*	1	0.17 (0.02–1.49)	0.16 (0.19–1.38)	1	0.12 (0.01–0.98)*	0.09 (0.01–0.74)*
7	2	0.51 (0.9–2.67)	0.26 (0.05–1.38)	0	–	–	1	0.44 (0.51–3.88)	0.31 (0.03–2.7)
Minimum Dietary Diversity		OR (95% CI)	AOR^a^ (95% CI)		OR (95% CI)	AOR^a^ (95% CI)		OR (95% CI)	AOR^a^ (95% CI)
YES (≥4 food groups)		Ref.	Ref.		Ref.	Ref.		Ref.	Ref.
NO (< 4 food groups)		1.19 (0.99–1.42)	1.37 (1.13–1.65)**		1.19 (0.83–1.71)	1.18 (0.82–1.69)		1.4 (1.08–1.79)**	1.49 (1.15–1.92)**

^**^P < .01, ^*^P < .05; ^a^Adjusted for age, gender, diarrhea and fever, CI confidence interval, OR Odds ratio, AOR Adjusted Odds ratio, Ref reference category. Both models fit the data equally well (all *P* > 0.10 in likelihood ratio test); *HAZ* Height-for-Age Z-scores, *WHZ* Weight-for-Height Z-scores, *WAZ* Weight-for-Age Z-scores, *SD* Standard deviation.

Table 3 shows unadjusted and adjusted odds from bivariate and multivariable logistic regression models for the association between specific food groups and stunting, wasting and underweight. The consumption of milk, meat and eggs has been found to be associated with reduced stunting among children. Children who did not consume any milk (AOR = 1.34; 95% CI; 1.09–1.63), meat (AOR = 1.27; 95% CI; 1.07–1.53), and eggs (AOR = 1.46; 95% CI; 1.05–2.03) have a higher likelihood of becoming stunted in the adjusted model. On the other hand, children who did not consume any grains had a higher likelihood of becoming wasted (AOR = 1.62; 95% CI; 1.02–2.58). Similarly, children who did not consume any legumes and nuts had a higher likelihood of becoming wasted in the unadjusted model (OR = 1.42; 95% CI; 1.0–1.96).

**Table 3:** Bivariate and multivariate logistic regression analysis of the association between food groups and stunting, wasting and underweight

Variable	Stunting (HAZ < -2SD)		Wasting (WHZ < -2SD)		Underweight (WAZ < -2SD)	
	Crude	Model^a^	Crude	Model^a^	Crude	Model^a^
	OR (95% CI)	AOR (95% CI)	OR (95% CI)	AOR (95% CI)	OR (95% CI)	AOR (95% CI)
FOOD GROUPS						
Grains roots and tubers						
YES	Ref.	Ref.	Ref.	Ref.	Ref.	Ref.
NO	0.89 (0.67–1.17)	1.05 (0.78–1.41)	1.59 (1.01–2.52)*	1.62 (1.02–2.58)*	1.22 (0.86–1.72)	1.34 (0.94–1.90)
Legumes and nuts						
YES	Ref.	Ref.	Ref.	Ref.	Ref.	Ref.
NO	0.9 (0.77–1.06)	0.96 (0.81–1.14)	1.4 (1.0–1.96)*	1.38 (0.98–1.94)	1.1 (0.87–1.35)	1.1 (0.89–1.39)
Milk and Dairy products						
YES	Ref.	Ref.	Ref.	Ref.	Ref.	Ref.
NO	1.38 (1.13–1.67)**	1.34 (1.09–1.63)**	1.26 (0.85–1.86)	1.24 (0.83–1.83)	1.26 (0.97–1.64)	1.22 (0.94–1.59)
Meat and flesh						
YES	Ref.	Ref.	Ref.	Ref.	Ref.	Ref.
NO	1.07 (0.91–1.26)	1.27 (1.07–1.53)**	1.0 (0.73–1.38)	0.97 (0.7–1.34)	0.98 (0.79–1.22)	1.04 (0.89–1.3)
Eggs						
YES	Ref.	Ref.	Ref.	Ref.	Ref.	Ref.
NO	1.37 (0.99–1.89)	1.46 (1.05–2.03)*	1.92 (0.89–4.15)	1.95 (0.9–4.23)	2.1 (1.24–3.52)**	2.1 (1.29–3.69)**
Vitamin A rich fruits and vegetables						
YES	Ref.	Ref.	Ref.	Ref.	Ref.	Ref.
NO	0.93 (0.79–1.1)	1.18 (0.99–1.41)	1.29 (0.95–1.76)	1.29 (0.94–1.78)	1.29 (1.04–1.59)*	1.46 (1.17–1.83)**
Other fruits and vegetables						
YES	Ref.	Ref.	Ref.	Ref.	Ref.	Ref.
NO	1.01 (0.83–1.22)	1.07 (0.88–1.31)	0.88 (0.61–1.26)	0.9 (0.63–1.29)	1.06 (0.82–1.37)	1.11 (0.86–1.44)

^**^P < .01, ^*^P < .05; ^a^Adjusted for age, gender, diarrhea and fever; *CI* confidence interval, *OR* Odds ratio, *AOR* Adjusted Odds ratio, *Ref* reference category, *HAZ* Height-for-Age Z-scores, *WHZ* Weight-for-Height Z-scores, *WAZ* Weight-for-Age Z-scores, *SD* Standard deviation

Moreover, the analysis shows that children who did not consume any egg or food made from eggs were more likely to be underweight. The likelihood of becoming underweight for children who did not consume any eggs is more than twice as high compared to children who consumed egg products (AOR = 2.1; 95%CI; 1.29–3.69). In addition, the likelihood of becoming underweight for children who did not consume vitamin A rich fruits and vegetables is higher compared to children who do (AOR = 1.46; 95% CI; 1.17–1.83).

## Discussion

This study aimed to examine the association between dietary diversity and undernutrition of children aged 6 to 23 months in Tanzania. In this study, dietary diversity was found to be a protective factor against stunting (HAZ) and underweight (WAZ) among children in Tanzania. These results are consistent with findings reported from other developing countries like Burkina Faso [19], Bangladesh [30], Ethiopia [18], and others [16, 21]. Our study suggests that undernutrition can be reduced by improving the dietary diversity of complementary foods in Tanzania. This is supported by the fact that dietary diversity is a good predictor of dietary quality and micronutrient density in children [15, 31]. Therefore, generally, this makes dietary diversity one of the important factors that policy makers should adopt to improve the nutritional status of children in the country.

However, this study did not find an association between wasting and the dietary diversity of children using the MDD indicator. This is in line with findings from other studies [19, 32]. As a previous study from Ethiopia has shown, wasting is more likely to be associated with diseases or household food insecurity rather than dietary diversity [18]. This might be due to the fact that wasting refers to acute malnutrition as a result of shorter-term episodes of inadequate feeding or illnesses [33].

Our study shows that only a small proportion (26%) of children of 6 to 23 months old in Tanzania reached the MDD. This is identical to a finding by Ochieng et al. who looked at factors affecting household nutrition security in Tanzania, which reported that only 26% of children had reached the MDD [27]. Overall, the present study shows that consumption of animal-source foods like meat, milk and eggs is not very common among children of 6–23 months in Tanzania. Similarly, the study by Ochieng et al. reported that meat and fish were consumed by less than 10% of children under five years [27]. These outcomes are not surprising considering the seasonal and geographical unavailability of some foods, the restrictions imposed by traditions, and possible financial constraints in Tanzania [34]. In this study, children who did not consume any meat were more likely to become stunted. This result corresponds with a survey of 12 to 59 month old children in Cambodia, which concluded that the consumption of animal-source foods was a protective factor against stunting and underweight [17]. Animal-source foods like meat, milk, eggs and poultry have a variety of micronutrients including vitamin A, vitamin B-12, riboflavin, calcium, iron and zinc that are difficult to obtain in adequate quantities from plant sourced foods alone [35]. Thus, insufficient intake of these nutrients may hinder the physical development of a child, resulting in stunting [2]. We therefore highly recommend that public health officials should educate parents and caregivers on the importance of animal-source foods for their children.

Moreover, our analysis emphasizes that the consumption of milk and dairy products is very beneficial for growing children. We found that non-consumption of milk products is a predictor of stunting among children. Failure to give any milk or dairy product at this critical age may result in protein imbalance [2]. Milk is one of the basic foods which can easily provide adequate nutrition when consumed regularly in relative small quantities and which can promote a child’s health and is available throughout the year [36]. Therefore, health education messages related to the importance of milk and dairy products as complementary foods is a critical public-health intervention in Tanzania.

Another interesting finding in this study was the association of vitamin A fruits and vegetables with underweight among children. In this study, children who did not consume vitamin A containing fruits and vegetables were more likely to become underweight. Similar findings were reported from a study among children in Ghana [21]. Also, previous research has shown a significant association between Vitamin A intake and undernutrition, but not among infants [37]. Vitamin A is known as an essential micronutrient for growth and immunity. Its deficiency is one of the most important causes of preventable childhood blindness and is a major contributor to morbidity and mortality from infections [37]. Therefore, based on these findings, lack of adequate vitamin A intake may results in the increase of underweight children in the country. It is therefore important for mothers to increase the inclusion of foods rich in vitamin A such as spinach, mangoes and papaya as complementary foods given to their children. These foods are relatively cheap in Tanzania and are easily accessible in both rural and urban areas for most of the year.

We also found that, children who did not consume any food made from grains or legumes were more likely to become wasted. In many countries, including Tanzania, grains and cereals including maize, sorghum, millet and rice are among the first foods that are introduced at the beginning of the complementary feeding period to infants [9, 22, 23, 38]. These are very beneficial for the children because they are an excellent source of energy, vitamins and minerals [38]. Therefore, to reduce the prevalence of undernutrition, mothers should provide adequate foods that provide adequate energy and all nutrients to their children.

However, it is important to mention some important limitation of this study. We only considered the dietary diversity as indicator of the overall quality of the child’s diet. This study did not take into account the quantity of the foods consumed, and is therefore not able to reliably predict that foods consumed met the required dietary intake. Also, due to the cross-sectional nature of this study, a cause and effect relationship cannot be assured and we have to interpret our findings with caution. Additionally, some indicators of the nutrition status like stunting represent a long-term cumulative process, whereas the dietary information available in the TDHS reflects only the previous day before the survey. In addition, the responses given by mothers/caregivers are sometime based on their ability to recall types of foods and their desire to please the surveyor. In spite of the mentioned limitations however, this present study can shed light on how dietary diversity is likely to influence the development of undernutrition in Tanzania. Future, large scale studies are needed to identify the causal relationship between diet and the physical and mental condition of the children in Tanzania.

## Conclusion

This study shows that consumption of a diverse diet is associated with a reduction in undernutrition among children of 6 to 23 months of age in Tanzania. In addition to dietary diversity, animal-source foods like meat, milk and eggs can prevent stunting in children. Especially the consumption of milk products is very important in order to reduce stunting among children. Measures to improve the type of complementary foods given to children to meet their needs for energy and nutrients should be considered. Moreover, strong commitment by the government and public health officials is needed to ensure adequate foods and education on what to feed are available.

## Data Availability

Data for this study are freely available upon request from the Demographic and health survey (DHS) portal (www.dhsprogram.com).

## Abbreviations

AOR: Adjusted Odd Ratio
CI: Confidence Intervals
FG: Food Group
HAZ: Height-for-Age Z-scores
IYCF: Infant and Young Child Feeding
MDD: Minimum Dietary Diversity
OR: Odd Ratio
SD: Standard Deviation
TDHS: Tanzania Demographic and Health Survey
WAZ: Weight-for-Age Z-scores
WHO: World Health Organisation
WHZ: Weight-for-Height Z-scores
